# Assessment of the prognostic value of the neutrophil-to-lymphocyte ratio and platelet-to-lymphocyte ratio in perihilar cholangiocarcinoma patients following curative resection: *A multicenter study of 333 patients*

**DOI:** 10.3389/fonc.2022.1104810

**Published:** 2023-01-04

**Authors:** Ming-Yang Ge, Zhi-Peng Liu, Yu Pan, Jiao-Yang Wang, Xiang Wang, Hai-Su Dai, Shu-Yang Gao, Shi-Yun Zhong, Xiao-Yu Che, Jing-Hua Zuo, Yun-Hua Liu, Xing-Chao Liu, Hai-Ning Fan, Wei-Yue Chen, Zi-Ran Wang, Xian-Yu Yin, Jie Bai, Yan-Qi Zhang, Yan Jiang, Yi Gong, Zhi-Yu Chen

**Affiliations:** ^1^ Department of Hepatobiliary Surgery, Southwest Hospital, Third Military Medical University (Army Medical University), Chongqing, China; ^2^ Department of Hepatobiliary Surgery, Sichuan Provincial People’s Hospital, Chengdu, China; ^3^ Department of Hepatobiliary Surgery, Affiliated Hospital of Qinghai University, Xining, China; ^4^ Clinical Research Center of Oncology, Lishui Hospital of Zhejiang University, Lishui, China; ^5^ Department of General Surgery, 903rd Hospital of People’s Liberation Army, Hangzhou, China; ^6^ Department of Health Statistics, College of Military Preventive Medicine, Third Military Medical University (Army Medical University), Chongqing, China

**Keywords:** neutrophil-to-lymphocyte ratio, platelet-to-lymphocyte ratio, perihilar cholangiocarcinoma, hepatectomy, survical

## Abstract

**Background & Aims:**

Tumor-associated chronic inflammation has been determined to play a crucial role in tumor progression, angiogenesis and immunosuppression. The objective of this study was to assess the prognostic value of the neutrophil-to-lymphocyte ratio (NLR) and platelet-to-lymphocyte ratio (PLR) in perihilar cholangiocarcinoma (pCCA) patients following curative resection.

**Methods:**

Consecutive pCCA patients following curative resection at 3 Chinese hospitals between 2014 and 2018 were included. The NLR was defined as the ratio of neutrophil count to lymphocyte count. PLR was defined as the ratio of platelet count to lymphocyte count. The optimal cutoff values of preoperative NLR and PLR were determined according to receiver operating characteristic (ROC) curves for the prediction of 1-year overall survival (OS), and all patients were divided into high- and low-risk groups. Kaplan-Meier curves and Cox regression models were used to investigate the relationship between values of NLR and PLR and values of OS and recurrence-free survival (RFS) in pCCA patients. The usefulness of NLR and PLR in predicting OS and RFS was evaluated by time-dependent ROC curves.

**Results:**

A total of 333 patients were included. According to the ROC curve for the prediction of 1-year OS, the optimal cutoff values of preoperative NLR and PLR were 1.68 and 113.1, respectively, and all patients were divided into high- and low-risk groups. The 5-year survival rates in the low-NLR (<1.68) and low-PLR groups (<113.1) were 30.1% and 29.4%, respectively, which were significantly higher than the rates of 14.9% and 3.3% in the high-NLR group (≥1.68) and high-PLR group (≥113.1), respectively. In multivariate analysis, high NLR and high PLR were independently associated with poor OS and RFS for pCCA patients. The time-dependent ROC curve revealed that both NLR and PLR were ideally useful in predicting OS and RFS for pCCA patients.

**Conclusions:**

This study found that both NLR and PLR could be used to effectively predict long-term survival in patients with pCCA who underwent curative resection.

## Introduction

Perihilar cholangiocarcinoma (pCCA) is a rare tumor that accounts for 50-70% of all biliary tract tumors and tends to occur at the site of biliary fusion or in the right or left liver duct (1, 2). Curative resection was the only treatment for achieving a potential cure, but long-term survival was poor (5-year survival ranged from 25% to 40%) (1, 3, 4). Accurate prediction of prognosis can better help surgeons develop personalized treatment strategies. However, the current prediction methods only include tumor markers and pathological tests. Assessment methods with more dimensions may enable more accurate prediction of patient prognosis.

Studies have shown that the tumor microenvironment and tumor-associated chronic inflammation play a crucial role in tumor progression (5, 6). During the development of pCCA, changes in inflammation levels further lead to immunosuppression and metabolic reprogramming and ultimately promote tumor progression. The condition of these patients could be reflected by complete blood count (CBC) markers (7), such as neutrophils, platelets, and lymphocytes. Specifically, neutrophils can directly promote tumor progression, metastasis and angiogenesis by releasing some enzymes (8–10). Platelets protect circulating tumor cells by encapsulating them in blood clots, protecting them from being lysed by natural killer cells or releasing thrombin to promote tumor proliferation and growth (11–13). Lymphocytes realize the tumor immune response through the recognition, killing and clearance of tumor cells, thereby playing a role in immunosuppression and antitumor immunity (14). Many studies have now confirmed that the inflammatory markers neutrophil-to-lymphocyte ratio (NLR) and platelet-to-lymphocyte ratio (PLR) (15–17) are associated with the prognosis of many cancers, such as hepatocellular carcinoma (HCC), intrahepatic cholangiocarcinoma, gallbladder carcinoma, and pancreatic cancer (18–22). However, the relationship between values of NLR and PLR and pCCA prognosis has not been studied.

The objective of this study was to assess the prognostic value of NLR and PLR in pCCA patients following curative resection. This was the first study conducted with data from a multicenter database on the long-term prognosis of NLR and PLR in pCCA patients undergoing curative resection.

## Methods

### Patient selection

Patients diagnosed with pCCA following curative resection between 2014 and 2018 at three hospitals in China (Southwest Hospital, Sichuan Provincial People’s Hospital, and Affiliated Hospital of Qinghai University) were included. The diagnosis of pCCA was confirmed by postoperative pathology. Patients who died within 30 days after surgery, those who had other autoimmune diseases, those who had inflammatory disease, or those whose data were missing important variables were excluded. Curative resection for this purpose included partial hepatic resection, cholangiotomy, biliary anastomosis, and lymph node dissection. If the tumor invaded the hepatic vein or hepatic artery, lateral vascular reconstruction was performed. Curative resection was defined as the resection of tissue with margins that were clear under the microscope without visible tumor cells. The study was approved by the Institutional Review Board of the Southwest Hospital of Chongqing, China (No. KY2021129). Because the study was retrospective and all data were anonymized, informed consent was not needed.

### Clinicopathological variables

The demographic variables included age, sex, American Society of Anesthesiologists (ASA) grade, preoperative jaundice, cirrhosis, chronic hepatitis, and hepatolithiasis. The laboratory variables included alanine aminotransferase (ALT), aspartate transaminase (AST), carbohydrate antigen 19-9 (CA19-9), total bilirubin (TB) and preoperative neutrophils, platelets and lymphocytes. The pathological variables included cirrhosis, maximum tumor size, nerve invasion, the 8th American Joint Committee on Cancer (AJCC) stage (23), the Bismuth classification (24), tumor differentiation, macro- or microvascular invasion, lymph node metastasis, and peripheral nerve invasion. Both portal vein invasion and hepatic artery invasion were considered macrovascular invasion. The operative variables included the extent of hepatectomy (major *vs*. minor), intraoperative blood loss, and perioperative blood transfusion.

For laboratory parameters, patients were divided into normal and abnormal groups using the upper or lower limit of the normal values used in clinical practice, such as 35 g/L for albumin, 40 U/L for AST, 40 U/L for ALT, 1 mg/dL TB, and 1.15 for INR, as reported in a previous study (25–28). Cirrhosis was confirmed by histopathological examination. Major hepatectomy was defined as the resection of three or more segments of the Couinaud liver, and minor hepatectomy was defined as the resection of fewer than three segments (29). Preoperative jaundice was defined as a preoperative total bilirubin higher than 34 μmol/L.

### Patient follow-up

Patients were followed-up after curative resection regularly. The postoperative surveillance strategy involved physical examination, abdominal ultrasonography and laboratory control every 2 months in the first and second years after resection, then once every three months from the third to the fifth year and finally once every six months. At each visit, tumor markers such as carcinoembryonic antigen (CEA) and CA19-9 were included, and computed tomography and/or magnetic resonance cholangiopancreatography examinations were also performed. Overall survival (OS) was computed as the interval between the date of surgery and the date of death or the last follow-up. The recurrence-free survival (RFS) was computed as interval from the day of resection to the day of diagnosis of tumor recurrence for recurrent patients or from the day of resection to the day of death or date of last follow-up for patients without recurrence.

### Statistical analysis

Continuous variables conforming to a normal distribution were expressed as the mean ± standard deviation and analyzed using *t test*s; those conforming to nonnormal distributions were expressed as the median (quartile) and tested with the Mann–Whitney U test. Categorical variables were expressed as numbers and percentages and compared between groups using the χ2 test or Fisher’s exact test. According to the ROC curve for the prediction of 1-year OS, the optimal cutoff values of preoperative NLR and PLR were calculated, and all patients were divided into high- and low-risk groups. RFS and OS were evaluated using the Kaplan–Meier method, and the differences between the two groups were examined by the log-rank test. Those variables with significance at *P* < 0.1 confirmed as noncollinearity by a variance inflation factor < 3 were entered into multivariable Cox proportional hazard models after univariable analyses, and 95% CI and hazard ratio values were calculated. The ability of NLR and PLR to predict OS and RFS was evaluated by time-dependent ROC curves. All data analyses were performed using SPSS software version 26.0 (IBM Corp., Armonk, NY, USA) and R software (version 3.5.1. http://www.r-project.org/). All *P* values reported were two-sided, and a *P* value < 0.05 was considered statistically significant.

## Results

### Clinicopathologic and operative variables of patients

**Figure 1:**
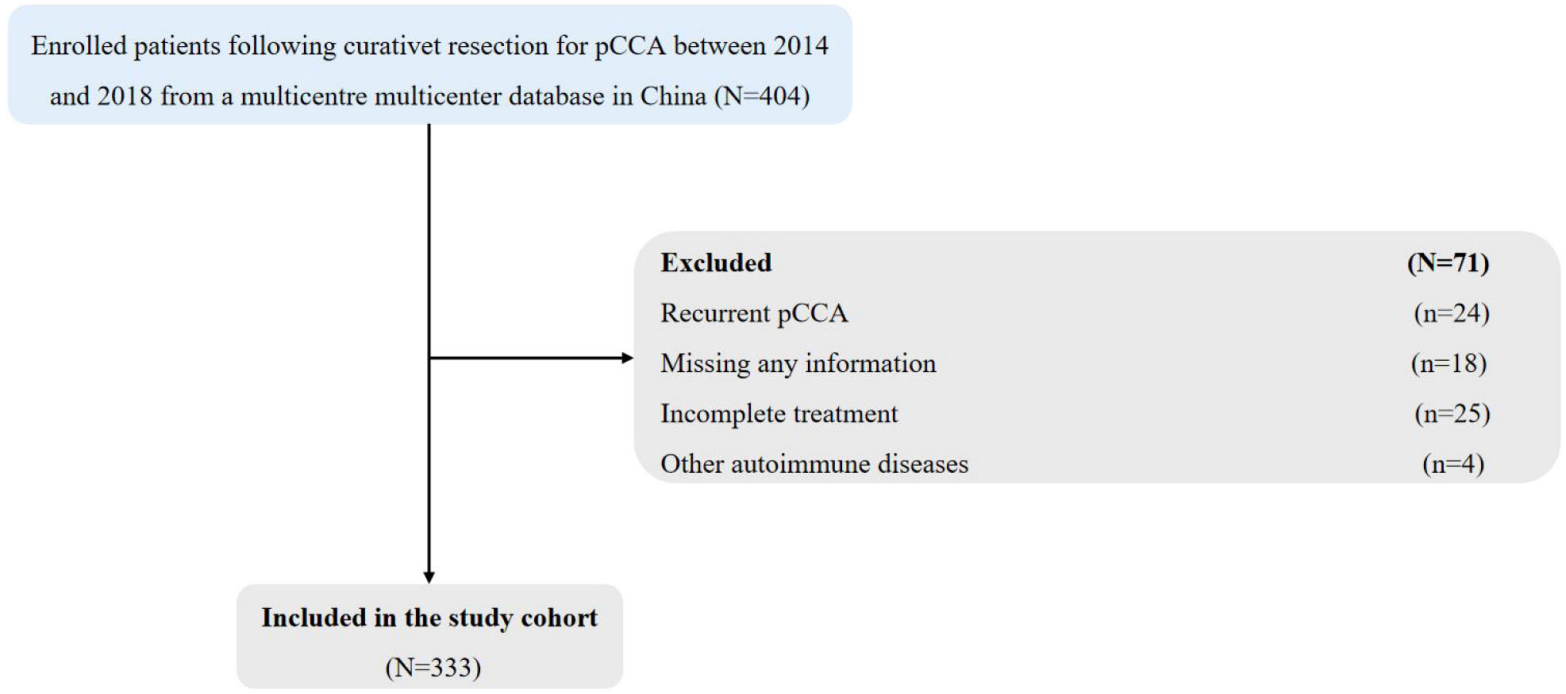
Flow chart of patient inclusion. pCCA, perihilar cholangiocarcinoma.

Among the 404 patients who underwent curative resection for pCCA between January 2014 and January 2018, we excluded 24 patients who had recurrent pCCA, 18 patients for whom information was missing, 26 patients with incomplete treatment, and 4 patients who had other autoimmune diseases as shown in **Figure 1**. Thus, 333 pCCA patients were included in the final analytic cohort (213 male and 120 female patients), and the mean age was 57.03 ± 9.94 years.

### ROC curves and cutoff values and groupings of NLR and PLR

**Figure 2:**
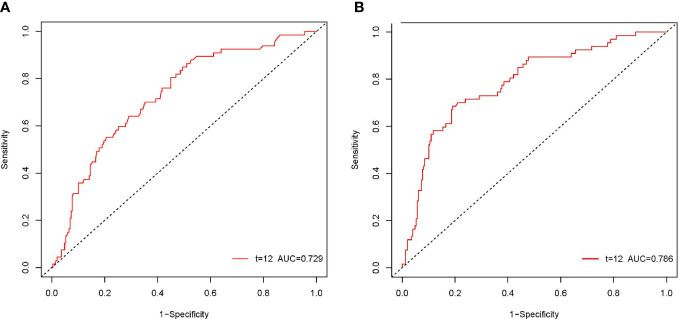
The ROC curves of the NLR and PLR in patients with pCCA. The ROC area of NLR was 0.729 **(A)**. The ROC area of PLR was 0.786 **(B)**.

According to the ROC curve, as shown in **Figure 2**, the optimal cutoff values of preoperative NLR and PLR for predicting 1-year OS were calculated to be 1.68 and 113.1, respectively. The ROC areas under the curve for NLR and PLR were 0.729 (95% CI: 0.663-0.795) and 0.786 (95% CI: 0.724-0.849), respectively. And according to their cutoff values, NLR < 1.68 was defined as low NLR (n = 155, 46.5%), NLR ≥ 1.68 was defined as high NLR (n =178, 53.5%), PLR < 113.1 (n = 231, 69.4%) was defined as low PLR, and PLR ≥ 113.1 was defined as high PLR (n = 102, 30.6%). The comparisons of patients’ clinicopathologic and operative variables between those with high and low NLR and PLR are shown in **Table 1**. High CA 19-9 level, poor differentiation and microvascular invasion were more commonly seen in high NLR patients (*P* < 0.05). Cirrhosis and lymph node metastasis were more commonly seen in high PLR patients (*P* < 0.05).

**Table 1:** Baseline for pCCA patients categorized by NLR, PLR and their clinical pathological characteristics.

Variables	NLR			PLR		
	< 1.68 (n = 155)	≥ 1.68 (n = 178)	*P* value	< 113.1 (n = 231)	≥113.1 (n = 102)	*P* value
Age, years*	56.89 ± 10.73	57.15 ± 9.22	0.815	56.51 ± 10.19	58.20 ± 9.29	0.154
Gender, male	101 (65.16)	112 (62.9)	0.671	149 (64.5)	64 (62.7)	0.758
ASA score > 2	12 (7.7)	22 (12.4)	0.165	26 (11.6)	8 (7.8)	0.343
Comorbidity	40 (25.8)	41 (23.0)	0.556	60 9 (26.0)	21 (20.6)	0.291
Preoperative jaundice	118 (76.1)	142 (79.8)	0.442	179 (77.5)	81 (79.4)	0.696
ALB (g/L)*	36.27 ± 4.71	37.25 ± 4.23	0.045	36.71 ± 4.70	37.00 ± 3.94	0.593
ALT (U/L)*	71.00 (45.50, 157.00)	83.50 (52.00, 169.00)	0.927	73.70 (46.00, 162.00)	81.15 (52.28, 161.00)	0.726
AST (U/L)*	74.00 (45.00, 136.00)	83.00 (52.75, 138.00)	0.735	75.40 (49.70, 130.00)	85.50 (49.95, 134.00)	0.665
Hb (g/L)	121.23 ± 25.51	122.75 ± 23.84	0.576	122.88 ± 27.76	120.15 ± 15.20	0.352
TB (mg/dL)*	150.40 (25.70, 279.40)	145.30 (51.95, 248.05)	0.557	150.40 (46.40, 263.90)	138.70 (32.40, 263.10)	0.839
CA 19-9 (U/L)*	121.00(34.20, 277.17)	140.07 (60.00, 364.65)	0.024	127.38 (42.53, 308.80)	134.52 (61.73, 400.00)	0.200
INR*	0.97 ± 0.10	0.97 ± 0.10	0.562	0.96 ± 0.10	0.98 ± 0.11	0.105
NLR	1.11 ± 0.32	3.15 ± 1.45	<0.001	1.76 ± 1.06	3.18 ± 1.81	<0.001
PLR	55.00 (38.92, 81.94)	107.27 (68.53, 144.45)	<0.001	60.00 (42.97, 81.25)	144.68 (126.88, 185.31)	<0.001
Cirrhosis	15 (9.7)	14 (7.8)	0.559	25 (10.8)	4 (3.9)	0.04
Chronic hepatitis	13 (9.0)	14 (7.8)	0.862	18 (7,8)	9 (8.8)	0.751
Hepatolithiasis	14 (8.3)	10 (5.6)	0.229	20 (8.7)	4 (3.9)	0.123
Maximum tumor size (cm)*	2.84 ± 1.24	3.05 ± 1.39	0.137	2.91 ± 1.26	3.05 ± 1.47	0.370
Poor differentiation	15 (9.7)	34 (19.1)	0.015	31 (13.4)	18 (17.6)	0.316
Macrovascular invasion	34 (21.9)	53 (29.8)	0.104	54 (23.4)	33 (32.4)	0.086
Microvascular invasion	11 (7.1)	26 (14.6)	0.030	23 (10.0)	14 (13.7)	0.313
8th AJCC stage III-IV	80 (51.6)	89 (50.0)	0.769	114 (49.4)	55 (53.9)	0.442
Bismuth classification III-IV	121 (78.1)	134 (75.3)	0.550	183 (79.2)	72 (70.6)	0.086
Lymph node metastasis	48 (30.1)	62 (34.8)	0.455	67 (29.0)	43 (42.1)	0.019
Peripheral nerve invasion	50 (32.3)	51 (28.7)	0.475	75 (32.5)	26 (25.5)	0.202
Intraoperative blood loss (ml)	700.0 (400.0, 1000.0)	700.0 (437.5, 1000.0)	0.137	700.0 (400.0, 1000.0)	700.0 (500.0, 1400.0)	0.116
Major hepatectomy	114 (73.5)	128 (71.9)	0.738	169 (73.2)	73 (71.6)	0.764

*Values are the mean ± standard deviation or median and quartile.

AJCC, American Joint Committee on Cancer; ALB, albumin level; ALT, alanine aminotransferase; ASA, American Society of Anesthesiologists; AST, aspartate transaminase; CA 19-9, carbohydrate antigen 19-9; INR, international normalized ratio; NLR, neutrophil-to-lymphocyte ratio; pCCA, perihilar cholangiocarcinoma; PLT, platelets level; PLR, platelet-to-lymphocyte ratio; TB, total bilirubin.

### Survival outcome

**Figure 3:**
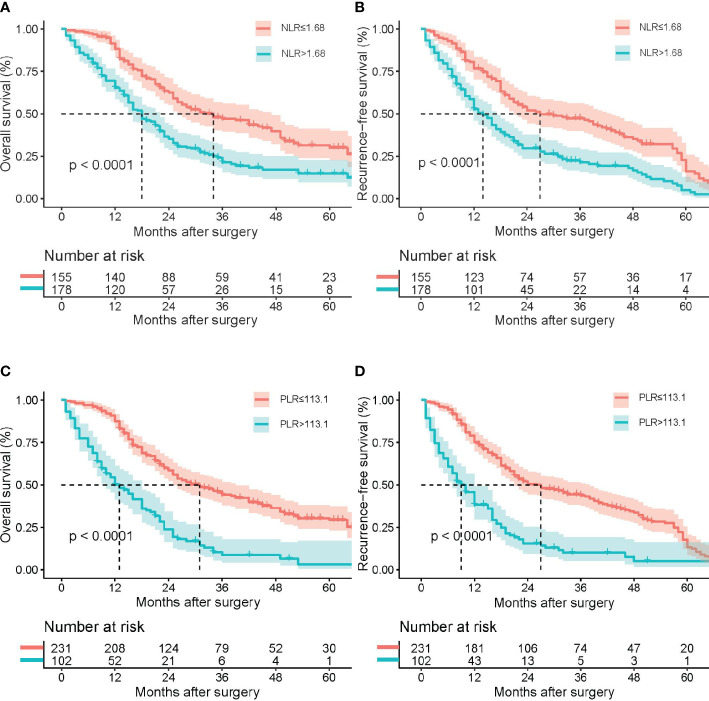
Kaplan–Meier survival analysis indicated that patients with NLR ≥ 1.68 had a shorter RFS and OS; patients with PLR *≥* 113.1 had a shorter RFS and OS. Overall survival **(A)** and recurrence-free survival **(B)** curve comparisons between patients with NLR ≥ 1.68 and NLR < 1.68; overall survival **(C)** and recurrence-free survival **(D)** curve comparisons between patients with PLR ≥ 113.1 and PLR < 113.1.

The median period of follow-up times, 5-year OS rates and 5-year RFS rates for all pCCA patients were 21.0 (12.0, 36.0) months, 22.0% and 10.5%, respectively. Regarding NLR, 5-year OS rates and 5-year RFS rates occurred for 14.9% and 5.2% in high NLR patients, respectively, and for 30.1% and 16.1% in low NLR patients, respectively. The rates of death and recurrence in high NLR patients were significantly lower in low NLR patients, as shown in **Table 2** (death, *P* = 0.004; recurrence, *P* = 0.084). Regarding PLR, 5-year OS rates and 5-year RFS rates occurred for 3.3% and 5.0% in high PLR patients, respectively, and for 29.4% and 13.3% in low PLR patients, respectively. The rates of death and recurrence in high PLR patients were significantly lower in low PLR patients, as shown in **Table 2** (death, *P* = 0.001; recurrence, *P* = 0.031). The survival and recurrence curves of high/low NLR patients and high/low PLR patients are shown in **Figure 3**.

**Table 2:** Comparisons of survival outcomes between pCCA patients with high and low NLRs and PLRs.

Survival outcomes	Total (n = 333)	NLR			PLR		
		< 1.68 (n = 155)	≥ 1.68 (n = 178)	*P* value	< 113.1 (n = 231)	≥ 113.1 (n = 102)	*P* value
Period of follow-up, months*	21.0 (12.0, 36.0)	27.0 (16.0, 49.0)	16.5 (8.8, 30.0)	<0.001	26.0 (15.0, 45.0)	12.0 (6.0, 22.3)	<0.001
Death during the follow-up	234 (70.3%)	97 (62.6%)	137 (77.0%)	0.004	146 (63.2%)	88 (86.3%)	0.001
Recurrence during the follow-up	267 (80.1%)	118 (76.1%)	149 (83.7%)	0.084	178 (77.1%)	89 (87.3%)	0.031
OS, months*	33.9 (30.8-37.2)	42.2 (37.4-46.9)	26.7 (22.6-30.8)	<0.001	40.7 (36.8-44.6)	17.8 (14.1-21.5)	<0.001
1-yr OS rate, %	76.1	88.3	65.4		87.4	50.3	
3-yr OS rate, %	33.7	47.1	21.4		44.3	8.7	
5-yr OS rate, %	22.0	30.1	14.9		29.4	3.3	
**RFS, months**	28.2 (25.5-30.9)	35.5 (31.4-39.6)	21.6 (18.4-24.7)	<0.001	33.9 (30.6-37.0)	15.0 (11.4-18.6)	<0.001
1-yr RFS rate, %	64.0	76.7	52.9		75.2	38.3	
3-yr RFS rate, %	33.9	47.4	21.5		43.8	10.1	
5-yr RFS rate, %	10.5	16.1	5.2		13.3	5.0	

*Values are the mean ± standard deviation or median and quartile.

OS, overall survival; RFS, recurrence-free survival; INR, international normalized ratio; NLR, neutrophil-to-lymphocyte ratio; pCCA, perihilar cholangiocarcinoma; PLR, platelet-to-lymphocyte ratio.

### NLR and PLR as prognostic markers

**Figure 4:**
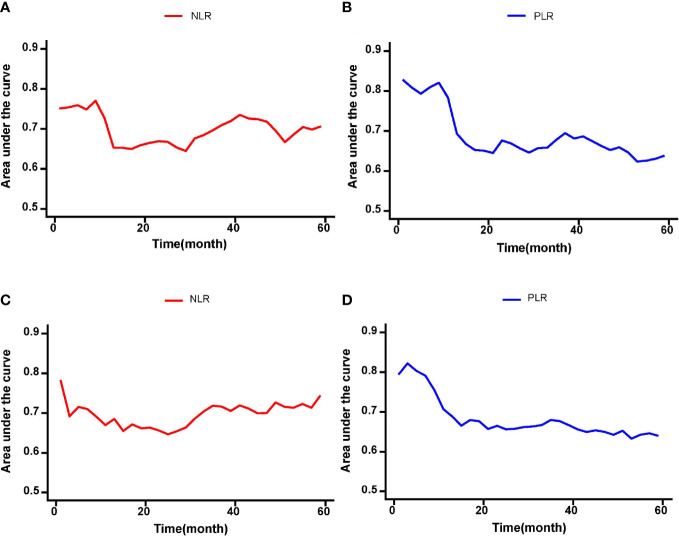
The time-dependent ROC curves of the NLR and PLR for OS and RFS in pCCA patients. **(A-D)** NLR and PLR were useful in effectively predicting long-term outcomes such as OS and RFS according to the results of time-dependent ROC analysis.

On multivariable Cox regression analyses, six variables were independently associated with OS in pCCA patients as shown in **Table 3**, including NLR < 1.68 *vs*. ≥ 1.68) (HR: 1.417, 95% CI: 1.071-1.875, P=); PLR (< 113.1 *vs*. ≥ 113.1) (HR: 2.223, 95% CI: 1.671-2.957); CA 19-9 (> 150 *vs*. ≤ 150 U/L) (HR: 1.610, 95% CI: 1.144-2.266); maximum tumor size (< 3 cm *vs*. ≥ 3 cm) (HR: 1.576, 95% CI: 1.204-2.062); macrovascular invasion (Yes *vs*. No) (HR: 1.416, 95% CI: 1.055-1.902); and lymph node metastasis (Yes *vs*. No) (HR: 2.012, 95% CI: 1.531-2.644). There were five independent variables associated with RFS in pCCA patients as shown in **Table 4**, including NLR (< 1.68 *vs*. ≥ 1.68) (HR: 1.598, 95% CI: 1.224-2.088); PLR (< 113.1 *vs*. ≥ 113.1) (HR: 2.138, 95% CI: 1.613-2.833), maximum tumor size (< 3 cm *vs*. ≥ 3 cm) (HR: 1.398, 95% CI: 1.080-1.812); macrovascular invasion (yes *vs*. no) (HR: 1.367, 95% CI: 1.030-1.815); and lymph node metastasis (yes *vs*. no) (HR: 1.638, 95% CI: 1.652-2.776). Moreover, NLR and PLR were found to be useful in effectively predicting OS and RFS through the result of the time-dependent ROC analysis, as shown in **Figure 4**.

**Table 3:** Univariable and multivariable Cox regression analyses for OS of pCCA patients.

Variables	Comparison	Univariable analyses		Multivariable analyses*	
		*P* value	HR (95% CI)	*P* value	HR (95% CI)
Age	> 60 *vs.* ≤ 60 years	0.470	1.101 (0.849-1.428)		
Gender	male *vs.* female	0.480	1.101 (0.842-1.440)		
ASA score	> 2 *vs.* ≤ 2	0.617	1.114 (0.729-1.702)		
Comorbidity	yes *vs.* no	0.191	1.217 (0.910-1.632)		
Preoperative jaundice	yes *vs.* no	0.193	1.221 (0.907-1.634)		
ALB	< 35 *vs.* ≥ 35 g/L	0.162	1.212 (0.926-1.586)		
ALT	> 40 *vs.* ≤ 40 U/L	0.263	1.215 (0.864-1.709)		
AST	> 40 *vs.* ≤ 40 U/L	0.505	1.120 (0.802-1.565)		
NLR*	< 1.68 *vs.* ≥ 1.68	< 0.001	1.941 (1.491-2.525)	0.015	1.417 (1.071-1.875)
PLR*	< 113.1 *vs.* ≥ 113.1	< 0.001	2.883 (2.196-3.783)	< 0.001	2.223 (1.671-2.957)
CA 19-9*	> 150 *vs*. ≤ 150 U/L	0.001	1.794 (1.278-2.516)	0.006	1.610 (1.144-2.266)
INR	> 1.15 *vs.* ≤ 1.15	0.211	1.398 (0.827-2.364)		
Cirrhosis	yes *vs.* no	0.612	1.123 (0.717-1.761)		
Chronic hepatitis	yes *vs.* no	0.181	0.720 (0.444-1.165)		
Hepatolithiasis	yes *vs.* no	0.431	1.202 (0.760-1.902)		
Maximum tumor size*	< 3 cm *vs.* ≥ 3 cm	< 0.001	1.735 (1.337-2.252)	0.001	1.576 (1.204-2.062)
Tumor differentiation*	poor *vs.* well/moderate	< 0.001	2.062 (1.491-2.883)	0.218	1.316 (0.851-2.305)
Macrovascular invasion*	yes *vs.* no	0.002	1.568 (1.191-2.065)	0.021	1.416 (1.055-1.902)
Microvascular invasion*	yes *vs.* no	< 0.001	2.201 (1.598-3.293)	0.051	1.620 (0.997-2.632)
Peripheral nerve invasion	yes *vs.* no	0.657	1.074 (0.811-1.413)		
Lymph node metastasis*	yes *vs.* no	< 0.001	2.138 (1.652-2.776)	< 0.001	2.012 (1.531-2.644)
Extent of hepatectomy	major *vs.* minor	0.213	1.207 (0.898-1.621)		

*Those variables found significant at P <.100 in univariable analyses were entered into multivariable Cox regression analyses.

ALB, albumin level; ALT, alanine aminotransferase; ASA, American Society of Anesthesiologists; AST, aspartate transaminase; CA19-9, carbohydrate antigen 19-9; CI, confidence interval; HR, hazard ratio; INR, international normalized ratio; NLR, neutrophil-to-lymphocyte ratio; pCCA, perihilar cholangiocarcinoma; PLT, platelets level; PLR, platelet-to-lymphocyte ratio; RFS, recurrence-free survival; TB, total bilirubin.

**Table 4:** Univariable and multivariable Cox regression analyses for RFS of pCCA patients.

Variables	Comparison	Univariable analyses		Multivariable analyses*	
		*P* value	HR (95% CI)	*P* value	HR (95% CI)
Age	> 60 *vs.* ≤ 60 years	0.115	1.011 (0.997-1.021)		
Gender	Male *vs.* Female	0.353	0.889 (0.692-1.139)		
ASA score	> 2 *vs.* ≤ 2	0.996	1.001 (0.779-1.281)		
Comorbidity	Yes *vs.* No	0.295	1.158 (0.878-1.541)		
Preoperative jaundice	Yes *vs.* No	0.199	1.982 (0.911-1.582)		
ALB	< 35 *vs.* ≥ 35 g/L	0.138	0.823 (0.641-1.072)		
ALT	> 40 *vs.* ≤ 40 U/L	0.077	1.326 (0.971-1.836)		
AST	> 40 *vs.* ≤ 40 U/L	0.350	1.162 (0.851-1.592)		
NLR*	< 1.68 *vs.* ≥ 1.68	<0.001	1.938 (1.511-2.479)	<0.001	1.598 (1.224-2.088)
PLR*	< 113.1 *vs.* ≥ 113.1	<0.001	2.772 (2.121-3.623)	<0.001	2.138 (1.613-2.833)
CA 19-9*	> 150 *vs*. ≤ 150 U/L	0.006	1.516 (1.132-2.047)	0.227	1.210 (0.888-1.649)
INR	> 1.25 *vs.* ≤ 1.25	0.503	1.192 (0.711-1.992)		
Cirrhosis	Yes *vs.* No	0.836	1.050 (0.671-1.639)		
Chronic hepatitis	Yes *vs.* No	0.375	0.822 (0.521-1.280)		
Hepatolithiasis	Yes *vs.* No	0.269	1.286 (0.821-2.005)		
Maximum tumor size*	< 3 cm *vs.* ≥ 3 cm	0.002	1.491 (1.162-1.912)	0.011	1.398 (1.080-1.812)
Tumor differentiation*	poor *vs.* well/moderate	<0.001	1.931 (1.403-2.659)	0.099	1.411 (0.937-2.124)
Macrovascular invasion*	Yes *vs.* No	0.001	1.592 (1.221-2.093)	0.031	1.367 (1.030-1.815)
Microvascular invasion*	Yes *vs.* No	<0.001	2.179 (1.531-3.108)	0.239	1.313 (0.835-2.065)
Peripheral nerve invasion	Yes *vs.* No	0.637	1.071 (0.822-1.389)		
lymph node metastasis*	Yes *vs.* No	<0.001	2.047 (1.593-2.629)	<0.001	1.638 (1.246-2.154)
Extent of hepatectomy	Major *vs.* Minor	0.784	1.039 (0.791-1.364)		

*Those variables found significant at P <.100 in univariable analyses were entered into multivariable Cox regression analyses.

ALB, albumin level; ALT, alanine aminotransferase; ASA, American Society of Anesthesiologists; AST, aspartate transaminase; CA19-9, carbohydrate antigen 19-9; CI, confidence interval; HR, hazard ratio; INR, international normalized ratio; NLR, neutrophil-to-lymphocyte ratio; pCCA, perihilar cholangiocarcinoma; PLR, platelet-to-lymphocyte ratio; PLT, platelet level; RFS, recurrence-free survival; TB, total bilirubin.

## Discussion

Cholangiocarcinoma represent a class of malignant tumors that originate in epithelial cells, of which hilar cholangiocarcinoma is the most common type, occurring at the site of biliary fusion or in the right or left liver duct (30) and accounting for approximately 50% of all cases. Resection is the only treatment with a potential of curing it. However, even after curative resection, the 5-year survival rate of patients is only 20% to 40% (1–3). Therefore, it is of great significance to actively identify prognostic factors that affect the long-term prognosis of pCCA. Many studies have demonstrated that chronic inflammation is associated with the occurrence and progression of tumors (5, 31). Studies have shown that cancer originates from chronic inflammatory sites, and there are a large number of inflammatory cells in tumor biopsies. Chronic inflammation provides a preferred microenvironment for the occurrence, progression and metastasis of tumors (5). During chronic inflammation, inflammatory cells and cytokines may act as tumor promoters, promoting cell survival, proliferation, invasion, and angiogenesis (5). Specific markers in the CBC panel of tests can be used as an accurate reflection of patients’ inflammatory levels, *via* generation of parameters such as NLR and PLR, and, thus, potentially assist clinicians in better predicting long-term prognosis in pCCA patients.

In previous studies, NLR and PLR have been shown to be important markers of long-term prognosis in patients with other digestive system tumors (18, 22). Hsiang et al. analyzed the long-term prognosis of 239 patients with hepatocellular carcinoma who underwent curative resection and found that the median OS of patients with NLR < 2.4 was significantly better than the median OS of patients with NLR ≥ 2.4 (median OS: 28.5 *vs*. 6.0 mo., *P* < 0.001). Sha et al. conducted a long-term prognostic analysis of 285 patients with gallbladder cancer who underwent cholecystectomy and found that the median OS of patients with NLR < 3.13 was significantly better than that of patients with NLR ≥ 3.13 (median OS: 13.0 *vs*. 8.27 mo., *P* < 0.001); the median OS of patients with PLR < 143.77 was significantly better than that of patients with PLR ≥ 143.77 (median OS: 10.80 *vs*. 10.27 mo., *P* > 0.05). However, the value of NLR and PLR in assessing long-term prognosis after curative resection of pCCA has not been demonstrated. To our knowledge, this is the first multicenter study to evaluate the usefulness of inflammatory markers NLR and PLR as indicators of OS and RFS after curative resection of pCCA.

In this multicenter study, a total of 333 patients underwent curative resection of pCCA. Moreover, the cutoff values obtained by NLR and PLR for predicting 1-year OS were used to group all patients, namely, the NLR cutoff values was 1.68, and the PLR cutoff values was 113.1. There were 178 patients (53.5%) in the high NLR group, 155 patients (46.5%) in the low NLR group, 102 patients (30.6%) in the high PLR group and 231 patients (69.4%) in the low PLR group. In the univariate analysis, the median OS and RFS of patients with a low NLR were significantly better than those with a high NLR (42.2 *vs*. 26.7 mo., *P* < 0.001; 35.5 *vs*. 21.6 mo., *P* < 0.001), and the median OS and RFS of patients with a low PLR were significantly better than those with a high PLR (40.7 *vs*. 17.8 mo., *P* < 0.001; 33.9 *vs*. 15.0 mo., *P* < 0.001). In multivariate Cox regression analysis, NLR and PLR were confirmed as independent risk factors for predicting OS and RFS in patients after curative resection of pCCA.

The mechanism of these phenomena has been confirmed and explained in previous studies (8, 9, 12, 13, 32–34). Neutrophils are the first responders to cell damage, and neutrophil infiltration marks persistent inflammation, which not only causes tissue damage but also, more importantly, promotes tumor progression, metastasis, and angiogenesis. In addition, reactive oxygen species and reactive nitrogen species produced by neutrophils can produce proto-oncogenes, leading to oxidative DNA damage and increasing genetic instability (8). It cannot be ignored that the enzymes produced and released by neutrophils, such as myeloperoxidase, neutrophil elastase, and matrix metalloproteinases, can also promote tumor progression (8). Neutrophils typically produce NETs (neutrophil-extracellular traps) during inflammation. NETs can capture circulating cancer cells (CTCs), and when they are released into the tumor microenvironment, they stimulate tumor cell migration and invasion (8, 9). Platelets also play an important role in promoting tumorigenesis and development. Platelets have been confirmed to promote tumor angiogenesis by releasing p-selectin and vascular endothelial growth factor, and they provide a suitable tumor microenvironment for tumor cell metastasis (12, 13). Platelets can also protect CTCs from being lysed by natural killer cells by encasing tumor cells in thrombi (32). However, as a type of immune cell, lymphocytes can play an important antitumor role. When the number of lymphocytes decreases, the body’s resistance to tumor cells decreases accordingly. Previous studies have reported that tumor-infiltrating lymphocytes play a positive role in resisting various advanced malignant tumors (33, 34).

In recent studies, there have been significant differences between the high NLR group and the low NLR group and between the high PLR group and the low PLR group. Before comparative analysis between the two groups, it may not be appropriate to use propensity score matching to examine the relationship between NLR, PLR and long-term oncology results to balance the baseline characteristics because this may lead to an increase in selection biases between the two groups. Therefore, univariate and multivariate Cox regression analyses were used in this study to determine whether high NLR and PLR were independently related to worse OS and RFS after curative resection of pCCA and to adjust for other prognostic risk factors.

With the advent of the era of immunotherapy, immune checkpoint inhibitors have achieved great success in the treatment of almost all solid tumors, bringing hope for tumor patients. Similarly, in related studies on biliary tumors, some clinical drug trials have achieved exciting progress (clinical trial information: NCT03875235 and NCT03875235) (35). In recent years, a PD-L1 receptor inhibitor (EnvafoLimab) has been approved by the FDA for the treatment of biliary tumors. Since both NLR and PLR can reflect immune function, clarifying the relationship between these two markers and prognosis will provide strong support for further exploring the formulation of personalized immunotherapy programs in patients with hilar cholangiocarcinoma.

This study has several limitations. First, it is a retrospective study, which will inevitably lead to bias in data collection. Second, the patients’ NLR and PLR values were calculated by a single measurement at admission, imposing some uncertainty. Third, the patients in this study were all from China, so data from Western patients was lacking. Therefore, the applicability of this conclusion to Western patients needs to be verified. Fourth, the number of patients included in this study was small; although it was a multicenter study, there were only 333 patients, possibly related to the relatively low incidence of pCCA. In the future, we will work with individual centers to provide a higher level of evidence.

In conclusion, our study suggests that NLR and PLR are potential prognostic factors for long-term prognosis in pCCA patients undergoing curative resection. After curative resection, these ratios are strongly correlated with survival, readily available, and economically determined. This study has strong clinical implications: higher NLR and PLR indicate a poor prognosis, so more attention should be given to patients with such values of NLR and PLR. It may be possible to minimize the levels of these two markers to improve the prognosis of patients, but further confirmation is needed. Once these findings have been validated in a larger prospective cohort, NLR and PLR markers could be used to help guide the clinical management of patients with pCCA.

## Data availability statement

The raw data supporting the conclusions of this article will be made available by the authors, without undue reservation.

## Ethics statement

The studies involving human participants were reviewed and approved by Southwest Hospital of Chongqing, China (No. KY2021129). The patients/participants provided their written informed consent to participate in this study.

## Author contributions

**Conception—**Z-YC, YJ, YG. **Study design—**M-YG, Z-PL, H-SD. **Administrative support—**Z-YC, YG. **Data collection and acquisition—**S-YG, S-YZ, X-YC, J-HZ, Y-HL, X-CL, H-NF, W-YC, Z-RW, X-YY, JB. **Data analysis—**M-YG, Z-PL, Y-QZ. **Manuscript preparation—**M-YG, Z-PL, YP, J-YW, XW. **Critical revision—**Z-YC, YJ, YG. **Final approval of manuscript—**All authors. M-YG, Z-PL, YP, J-YW, and XW contributed equally to this work. All authors contributed to the article and approved the submitted version.

## References

[1] Ito F Cho CS Rikkers LF Weber SM. Hilar cholangiocarcinoma: Current management. Ann Surg (2009) 250(2):210–8. doi: 10.1097/SLA.0b013e3181afe0ab 19638920

[2] Soares KC Jarnagin WR. The landmark series: Hilar cholangiocarcinoma. Ann Surg Oncol (2021) 28(8):4158–70. doi: 10.1245/s10434-021-09871-6 PMC9273057 33829358

[3] Soares KC Kamel I Cosgrove DP Herman JM Pawlik TM. Hilar cholangiocarcinoma: Diagnosis, treatment options, and management. Hepatobiliary Surg Nutr (2014) 3(1):18–34. doi: 10.3978/j.issn.2304-3881.2014.02.05 24696835 PMC3955000

[4] Liu ZP Zhang QY Chen WY Huang YY Zhang YQ Gong Y. Evaluation of four lymph node classifications for the prediction of survival in hilar cholangiocarcinoma. J Gastrointest Surg (2022) 26:1030–40. doi: 10.1007/S11605-021-05211-X PMC9085675 34973138

[5] Zhao H Wu L Yan G Chen Y Zhou M Wu Y Li Y. Inflammation and tumor progression: Signaling pathways and targeted intervention. Signal Transd Target Ther (2021) 6(1):263. doi: 10.1038/s41392-021-00658-5 PMC8273155 34248142

[6] Zhou J Valentini E Boutros M. Microenvironmental innate immune signaling and cell mechanical responses promote tumor growth. Dev Cell (2021) 56(13):1884–1899.E5. doi: 10.1016/j.devcel.2021.06.007 34197724

[7] Rojko L Megyesfalvi Z Czibula E Reiniger L Teglasi V Szegedi Z. Longitudinal analysis of complete blood count parameters in advanced-stage lung cancer patients. Thorac Cancer (2020) 11:3193–204. doi: 10.1111/1759-7714.13642 PMC7605999 32941706

[8] Giese MA Hind LE Huttenlocher A. Neutrophil plasticity in the tumor microenvironment. Blood (2019) 133(20):2159–67. doi: 10.1182/blood-2018-11-844548 PMC6524564 30898857

[9] Masucci MT Minopoli M Del Vecchio S Carriero MV. The emerging role of neutrophil extracellular traps (Nets) in tumor progression and metastasis. Front Immunol (2020) 11:1749. doi: 10.3389/fimmu.2020.01749 33042107 PMC7524869

[10] Hedrick CC Malanchi I. Neutrophils in cancer: Heterogeneous and multifaceted. Nat Rev Immunol (2022) 22(3):173–87. doi: 10.1038/s41577-021-00571-6 34230649

[11] Menter DG Tucker SC Kopetz S Sood AK Crissman JD Honn KV. Platelets and cancer: A casual or causal relationship: Revisited. Cancer Metastasis Rev (2014) 33(1):231–69. doi: 10.1007/s10555-014-9498-0 PMC4186918 24696047

[12] Schlesinger M. Role of platelets and platelet receptors in cancer metastasis. J Hematol Oncol (2018) 11(1):125. doi: 10.1186/s13045-018-0669-2 30305116 PMC6180572

[13] Haemmerle M Stone RL Menter DG Afshar-Kharghan V Sood AK. The platelet lifeline to cancer: Challenges and opportunities. Cancer Cell (2018) 33(6):965–83. doi: 10.1016/j.ccell.2018.03.002 PMC5997503 29657130

[14] Meyer CE Key PN Zhu T Shabsovich M Ni A Tripathy SK. Expression of the inhibitory receptor Nkg2a correlates with increased liver and splenic nk cell response to activating receptor engagement. Immun Inflammation Dis (2017) 5(2):177–89. doi: 10.1002/iid3.156 PMC5418142 28474506

[15] Chen Y Yang Y Zhang XY Fan QS Li X Xin YJ. Nomogram based on neutrophil-To-Lymphocyte ratio and platelet-To-Lymphocyte ratio to predict recurrence in patients with hepatocellular carcinoma after radiofrequency ablation. Cardiovasc Intervent Radiol (2021) 44(10):1551–60. doi: 10.1007/s00270-021-02872-8 34036405

[16] Diem S Schmid S Krapf M Flatz L Born D Jochum W. Neutrophil-To-Lymphocyte ratio (Nlr) and platelet-To-Lymphocyte ratio (Plr) as prognostic markers in patients with non-small cell lung cancer (Nsclc) treated with nivolumab. Lung Cancer (2017) 111:176–81. doi: 10.1016/J.Lungcan.2017.07.024 28838390

[17] Fang T Wang Y Yin X Zhai Z Zhang Y Yang Y. Diagnostic sensitivity of nlr and plr in early diagnosis of gastric cancer. J Immunol Res (2020) 2020:9146042. doi: 10.1155/2020/9146042 32211444 PMC7081040

[18] Zhu S Yang J Cui X Zhao Y Tao Z Xia F. Preoperative platelet-To-Lymphocyte ratio and neutrophil-To-Lymphocyte ratio as predictors of clinical outcome in patients with gallbladder cancer. Sci Rep (2019) 9(1):1823. doi: 10.1038/s41598-018-38396-4 30755649 PMC6372648

[19] Toledano-Fonseca M Cano MT Inga E Gómez-España A Guil-Luna S García-Ortiz MV. The combination of neutrophil-lymphocyte ratio and platelet-lymphocyte ratio with liquid biopsy biomarkers improves prognosis prediction in metastatic pancreatic cancer. Cancers (Basel) (2021) 13(6):5. doi: 10.3390/cancers13061210 PMC7998484 33802006

[20] Muhammed A Fulgenzi C Dharmapuri S Pinter M Balcar L Scheiner B. The systemic inflammatory response identifies patients with adverse clinical outcome from immunotherapy in hepatocellular carcinoma. Cancers (Basel) (2021) 14(1):6–11. doi: 10.3390/cancers14010186 PMC8750517 35008350

[21] Sellers CM Uhlig J Ludwig JM Stein SM Kim HS. Inflammatory markers in intrahepatic cholangiocarcinoma: Effects of advanced liver disease. Cancer Med (2019) 8(13):5916–29. doi: 10.1002/cam4.2373 PMC6792510 31429524

[22] Hsiang CW Huang WY Yang JF Shen PC Dai YH Wang YF. Dynamic changes in neutrophil-To-Lymphocyte ratio are associated with survival and liver toxicity following stereotactic body radiotherapy for hepatocellular carcinoma. J Hepatocell Carcinoma (2021) 8:1299–309. doi: 10.2147/JHC.S334933 PMC8573140 34765571

[23] Chun YS Pawlik TM Vauthey JN. 8th edition of the ajcc cancer staging manual: Pancreas and hepatobiliary cancers. Ann Surg Oncol (2018) 25(4):845–7. doi: 10.1245/s10434-017-6025-x 28752469

[24] Bismuth H Nakache R Diamond T. Management strategies in resection for hilar cholangiocarcinoma. Ann Surg (1992) 215(1):31–8. doi: 10.1097/00000658-199201000-00005 PMC1242367 1309988

[25] Liu ZP Chen WY Wang ZR Liu XC Fan HN Xu L. Development and validation of a prognostic model to predict recurrence-free survival after curative resection for perihilar cholangiocarcinoma: A multicenter study. Front Oncol (2022) 12:849053. doi: 10.3389/fonc.2022.849053 35530316 PMC9071302

[26] Liu ZP Chen WY Zhang YQ Jiang Y Bai J Pan Y. Postoperative morbidity adversely impacts oncological prognosis after curative resection for hilar cholangiocarcinoma. World J Gastroenterol (2022) 28(9):948–60. doi: 10.3748/wjg.v28.i9.948 PMC8908289 35317056

[27] Liu ZP Cheng ZJ Dai HS Zhong SY Zhao DC Gong Y. Impact of perioperative blood transfusion on long-term survival in patients with different stages of perihilar cholangiocarcinoma treated with curative resection: A multicentre propensity score matching study. Front Oncol (2022) 12:1059581. doi: 10.3389/Fonc.2022.1059581 36387093 PMC9660252

[28] Liu ZP Yao LQ Diao YK Chen ZX Feng ZH Gu WM. Association of preoperative body mass index with surgical textbook outcomes following hepatectomy for hepatocellular carcinoma: A multicenter study of 1206 patients. Ann Surg Oncol (2022) 3-4. doi: 10.1245/S10434-022-11721-Y 35419755

[29] Adam R Chiche L Aloia T Elias D Salmon R Rivoire M. Hepatic resection for noncolorectal nonendocrine liver metastases: Analysis of 1,452 patients and development of a prognostic model. Ann Surg (2006) 244:524–35. doi: 10.1097/01.Sla.0000239036.46827.5f PMC1856551 16998361

[30] Valle JW Kelley RK Nervi B Oh DY Zhu AX. Biliary tract cancer. Lancet (2021) 397(10272):428–44. doi: 10.1016/S0140-6736(21)00153-7 33516341

[31] Coussens LM Werb Z. Inflammation and cancer. Nature (2002) 420(6917):860–7. doi: 10.1038/nature01322 PMC2803035 12490959

[32] Nieswandt B Hafner M Echtenacher B Männel DN. Lysis of tumor cells by natural killer cells in mice is impeded by platelets. Cancer Res (1999) 59(6):1295–300. 10096562

[33] Topalian SL Solomon D Avis FP Chang AE Freerksen DL Linehan WM. Immunotherapy of patients with advanced cancer using tumor-infiltrating lymphocytes and recombinant interleukin-2: A pilot study. J Clin Oncol (1988) 6(5):839–53. doi: 10.1200/JCO.1988.6.5.839 3259261

[34] Restifo NP Dudley ME Rosenberg SA. Adoptive immunotherapy for cancer: Harnessing the T cell response. Nat Rev Immunol (2012) 12(4):269–81. doi: 10.1038/nri3191 PMC6292222 22437939

[35] Collingridge D. Asco annual meeting. Lancet Oncol (2022) 23:844. doi: 10.1016/S1470-2045(22)00338-2 35691299

